# Morphological Study of the Alimentary Canal and Malpighian Tubules in the Adult of the Pollen Beetle *Meligethes* (*Odonthogethes*) *chinensis* (Coleoptera: Nitidulidae: Meligethinae)

**DOI:** 10.3390/insects14030298

**Published:** 2023-03-20

**Authors:** Longyan Chen, Meike Liu, Andrea Di Giulio, Xinxin Chen, Simone Sabatelli, Wenkai Wang, Paolo Audisio

**Affiliations:** 1 Institute of Entomology, College of Agriculture, Yangtze University, Jingzhou 434025, China; clylyly@126.com (L.C.); wakeychen@163.com (X.C.); 2 Department of Science—L.I.M.E., University of Roma Tre, 00146 Rome, Italy; andrea.digiulio@uniroma3.it; 3 Department of Biology and Biotechnology, Sapienza Rome University, 00185 Rome, Italy; simone.sabatelli@uniroma1.it (S.S.); paolo.audisio@uniroma1.it (P.A.); 4 MARA Key Laboratory of Sustainable Crop Production in the Middle Reaches of the Yangtze River (Co-Construction by Ministry and Province), College of Agriculture, Yangtze University, Jingzhou 434025, China; w_wenkai@hotmail.com

**Keywords:** digestive system, excretory system, SEM microscopy, functional morphology

## Abstract

**Simple Summary:**

Meligethinae has entirely become strictly anthophagous, also being called “pollen beetles”, with all members (~700 species) of this subfamily using pollen and other floral parts as food resources for their larvae and adults. In this study, we used light, fluorescence, and scanning electron microscopy (LM, FM, and SEM) to explore the fine morphological structure of the alimentary canal and Malpighian tubules of *Meligethes* (*Odonthogethes*) *chinensis*, a common Chinese pollen beetle associated with flowers of Rosaceae. The results show that the alimentary canal of *M*. (*O*.) *chinensis* is divided into three parts of foregut, midgut, and hindgut. The foregut is the shortest part and has no crop; the midgut is the widest part with numerous blunt-fingered gastric ceca; the front of the hindgut folds in a circle and then extends back to the anus. Six Malpighian tubules are attached to the colon to form a cryptonephridial system. We also provide a schematic color picture of the alimentary canal and Malpighian tubules in the hemocoelic cavity of the dissected *M*. (*O*.) *chinensis*. This study is the first to systematically study the general morphology of the alimentary canal and Malpighian tubules of Meligethinae, which can provide important support for subsequent anatomical and physiological studies of anthophagous beetles.

**Abstract:**

*Meligethes* (*Odonthogethes*) *chinensis* is a highly specialized species of Nitidulidae in China that takes pollen as its main food source, and its main host plant is *Rubus idaeus* L. (Rosaceae). In this study, the structural morphology of the alimentary canal and Malpighian tubules of adult *M*. (*O*.) *chinensis* was observed under light, fluorescence, and scanning electron microscopy. The alimentary canal of adult *M*. (*O*.) *chinensis* is divided into foregut, midgut, and hindgut. The foregut is the shortest and consists of the pharynx, esophagus, proventriculus, and cardiac valve. The midgut is a straight, distended, cylindrical, thin-walled tube. Numerous blunt-fingered gastric ceca are distributed irregularly throughout the midgut. The hindgut is subdivided into the ileum, colon, and rectum. The ileum is coiled. The colon gradually enlarges posteriorly. The rectum is thickly muscled and followed by a membranous structure. The openings of proximal Malpighian tubules are evenly inserted into the junction of the midgut and hindgut, and distal Malpighian tubules are evenly attached to the colon to form a cryptonephridial system. In this study, we also compare the structure and infer the function of the alimentary canal and Malpighian tubules among beetles, as well as discuss the evolutionary and taxonomical implications.

## 1. Introduction

Insects gained new food sources after radiation of flowering angiosperms in the Cretaceous, and new feeding strategies evolved correspondingly. Then, most groups of flower-visiting beetles appeared successively [1,2]. Beetles (Coleoptera), one of the oldest and most diverse groups of insects, visited flowers before the emergence of butterflies and bees [3,4]. Flower-visiting beetles are important pollinators [5,6,7]. They can carry pollen to help plants pollinate when they fly. In return, plants usually provide them with petals, nutrient-rich pollen, and nectar as food [8,9,10,11]. Meligethinae is (in company with members of the only moderately related neotropical tribe Mystropini in the subfamily Nitidulinae) the only independent subfamily in Nitidulidae, wholly associated with flowers for larval development, with some 700 species in the world. Meligethinae, in fact, has entirely become strictly anthophagous, also being called “pollen beetles”, with all members of this subfamily using pollen and other floral parts as food resources for their larvae and adults. The subfamily Meligethinae represents an important group for studying the relationship between specialized phytophagous insects and their host plants [12,13,14,15,16,17,18,19,20,21,22,23,24,25,26,27].

The alimentary canal of insects is a simple straight or coiled continuous tube that extends from the mouthparts to the anus at the end of the abdomen [28]. It is the organ that ingests, transports, digests food, and absorbs nutrients. It also has the functions of controlling the water balance, excretion, and other functions. Insects have different feeding strategies, and the morphology and structure of their alimentary canals also show some differences, reflecting their adaptability to the environment and food [29,30]. Generally, the alimentary canal of Coleoptera is divided into three parts: the foregut, the midgut, and the hindgut [31]. The foregut originates from the ectoderm. It consists of the pharynx, esophagus, crop, and proventriculus and cardiac valve. Some Adephaga beetles (such as many Carabidae and some Dytiscidae) generally have a large crop formed by the dilated esophagus that is used for the temporary storage of food. In most polyphagous beetles, the crop is absent or only weakly developed [32,33,34]; however, well-developed crops exist in some species in Curculionidae and Chrysomelidae [32,35]. Especially for pollen-eating beetles, crops seem to play an important role in dealing with the pollen grains that are quite tough and impermeable. At least in Oedemeridae, there is a lateral sac-like diverticulum with densely packed pollen grains, and it has been found that pollen grains may germinate in this crop diverticulum, which may also be a characteristic of this family [33,36]. The foregut mainly has the functions of receiving, transporting, grinding, filtering, and initially digesting food [37,38]. The midgut originates from the entoderm and is the main place for food digestion and nutrient absorption [39], especially in maintaining ion balance and water transport [34,40]. The surface of the midgut is sometimes smooth and uniform, sometimes with saccate protrusions, which are globular, filiform elongate, or finger-like papillae, and it varies in position, number, arrangement, and nomenclature among different species and groups [33,41,42,43,44,45,46,47,48]. The hindgut originates from the ectoderm and is divided into three parts: the ileum, colon, and rectum [49]. It plays an important role in the temporary storage and the ejection of food remnants, as well as the reabsorption of water and minerals from the feces before excretion [50,51]. Malpighian tubules are important parts of excretory organs; they also originate from the ectoderm [52]. Most Coleoptera have four or six Malpighian tubules. The openings of the Malpighian tubules insert into the junction of the midgut and hindgut. Proximal tubules are free in the hemocoelic cavity, and distal tubules wander in the hemocoelic cavity or form a cryptonephridial system with the hindgut [33,43,47,53,54,55,56,57,58]. Malpighian tubules are usually arranged in one of two ways: (1) evenly around the alimentary canal; (2) in groups of one or two or three around the alimentary canal [32,42,52,53].

The evolution of pollen feeding habits of pollinating beetles has attracted increasing attention [4,59,60,61,62,63]. However, the morpho-functional adaptability of Meligethinae, which feeds exclusively on pollen, has not been studied yet. Only Stammer (1934) gave a brief description of the distribution of the Malpighian tubules of the genus *Meligethes* [52]. In fact, the subfamily Meligethinae exhibits a variety of morphological diversity and adaptations, advanced ecological specializations, and highly specialized insect–host plant interactions. The level of these adaptations is higher than in all other subfamilies among Nitidulidae. This set of adaptations can, therefore, also be used to depict more general scenarios in the study of the evolution of the anthophagy. *Meligethes* (*Odonthogethes*) *chinensis* Kirejtshuk, 1979, is a common and widespread Chinese species, associated as larvae with *Rubus* spp. (Rosaceae), with its main larval host plant being *Rubus idaeus* L. [64,65]; this species was the target of the present study. We used light, fluorescence, and scanning electron microscopy to reveal the morphological structure of the alimentary canal and Malpighian tubules of this species, speculated on the function of crop, proventriculus, midgut “saccate protrusions”, cryptonephridial system, and Malpighian tubules, and we discuss their potential applications in taxonomy and evolution, to provide evidence for the study of Meligethinae’s pollen-feeding strategy and coevolution with host plants.

## 2. Materials and Methods

### 2.1. Specimens

The adult specimens of *Meligethes* (*Odonthogethes*) *chinensis* used or analyzed in this study were collected from flowering *Rubus idaeus* (Rosaceae) in Shennongjia Forestry District, Hubei Province, China in June 2021, and then placed in plastic freezing tubes and stored in a refrigerator at −20 °C until dissection.

### 2.2. Light Microscopy (LM) Research

Specimens from 10 males and 10 females were transferred to a 4 °C refrigerator to thaw; then, they were dissected on a glass slide and observed using a Nikon SMZ 1500 microscope. We used a No. 5 cell clamp to remove the hard pronotum and elytra, used a No. 00 insect needle to cut intersegmental membranes, and carefully peeled off tergites to expose the internal organs. Then, we used a small hook hair to carefully peel off the muscles, fat bodies, and other tissues and organs around the alimentary canal and Malpighian tubules. After rinsing three times in PBS solution, pictures were taken using a DFC 450 camera connected to a Leica M205A electrodynamic stereo microscope.

### 2.3. Fluorescence Microscopy (FM) Research

After dissection, the alimentary canal and Malpighian tubules from 10 males and 10 females were quickly transferred into 4% paraformaldehyde for 2 h. After rinsing three times in 0.01 M PBS (pH 7.2–7.4), we transferred them to 2% Triton X-100 for 0.5 h. After rinsing three times in 0.01 M PBS, we stained them with 2% aniline blue staining solution for 5 min. Next, we rinsed them three times in 0.01 M PBS, before finally transferring the alimentary canal and Malpighian tubules to a glass slide dripped with 50% glycerol, and then sealing the cover glass. Lastly, we observed and photographed the prepared material using a Nikon Eclipse 80i fluorescence microscope.

### 2.4. Scanning Electron Microscope (SEM) Research

The alimentary canal and Malpighian tubules from 10 males and 10 females were frozen in 2.5% glutaraldehyde for 12 h at 4 °C, rinsed five times in 0.01 M PBS for 20 min each, dehydrated by graded ethanol (30% 10 min, 50% 10 min, 70% 10 min, 80% 10 min, 90% 20 min, 95% 25 min, and 100% 30 min), and then washed with a mixture of absolute ethanol and *tert*-butanol with volume ratios of 3:1, 1:1, and 1:3 (*v*/*v*) for 15 min each. Subsequently, they were treated with tert-butanol twice for 20 min each, dried in a freeze dryer (Christ-Alpha 1-2 LD) for 2 h, sprayed gold in an ion sputter coater (Quorum-SC7620), and finally observed and documented under a scanning electron microscope (Tescan-Vega 3 SBU) at 20 kV.

### 2.5. Data Analysis

We randomly selected and dissected 10 males and 10 females of *M*. (*O*.) *chinensis* to obtain the alimentary canals. The lengths of each adult and the alimentary canal, as well as the foregut, midgut, and hindgut, were measured separately. The width of each part of the alimentary canal (esophagus, proventriculus, midgut, ileum, colon, and rectum) was measured separately, and Shapiro–Wilk and Levene’s tests were used to assess the normality and homogeneity of variance, respectively. When the data were normally distributed and the variances were homogeneous, Student’s *t*-test (*p* < 0.05) was used; otherwise, the Mann–Whitney test (*p* < 0.001) was used to judge whether the difference was significant with SPSS 26.0 (http://www.spss.com, accessed on 1 August 2022) [66].

Line drawings were completed with Adobe Illustrator 2020. Pictures were processed with Photoshop CS 6.0. Terminologies mainly referred to Snodgrass (1935) and Candan et al. (2020) [31,43,58]. Some abbreviations used in this study are presented below.
An: anusHg: hindgutPh: pharynxCm: circular muscleIl: ileumPl: pylorus regionCo: colonLbr: labrumPv: proventriculusCry: cryptonephridial systemLm: longitudinal muscleRc: rectumCv: cardiac valveMb: muscle bundleSe: setaeEp: epitheliumMg: midgutTl: tracheoleEs: esophagusMl: muscle layerTm: transverse muscleFg: foregutMp: mouthpartTr: tracheaGc: gastric cecumMt (1, 2, 3, 4, 5, 6): Malpighian tubule (1, 2, 3, 4, 5, 6)

## 3. Results

### 3.1. General Morphology of Alimentary Canal and Malpighian Tubules

**Figure 1:**
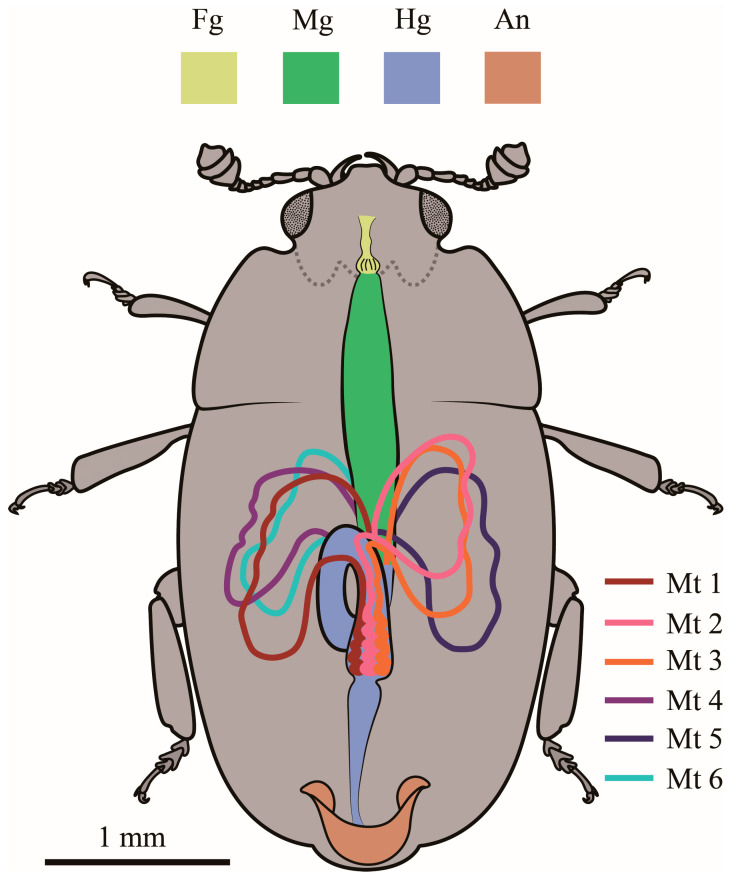
Schematic color picture (in dorsal view) of the dissected *Meligethes* (*Odonthogethes*) *chinensis*, showing the distribution of the alimentary canal and Malpighian tubules in the hemocoelic cavity. Fg: foregut, Mg: midgut, Hg: hindgut, An: anus, Mt: Malpighian tubule.

**Figure 2:**
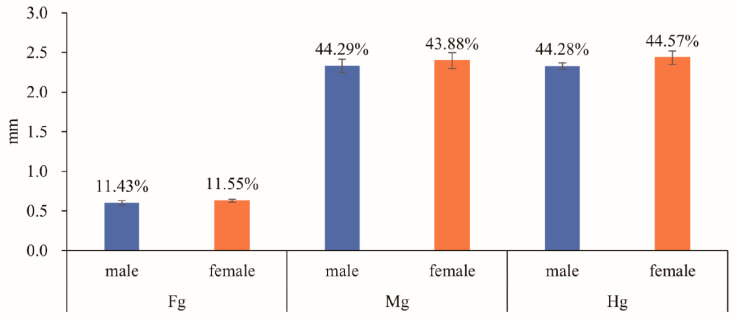
Relative proportions (mean ± SE) of three regions (Fg: foregut, Mg: midgut, Hg: hindgut) of the alimentary canal of male and female *M*. (*O*.) *chinensis* (*n* = 10). The y-axis (mm) indicates the actual length of each part of the alimentary canal; the numbers on the bar chart represent the percentage of each part of the alimentary canal.

**Figure 3:**
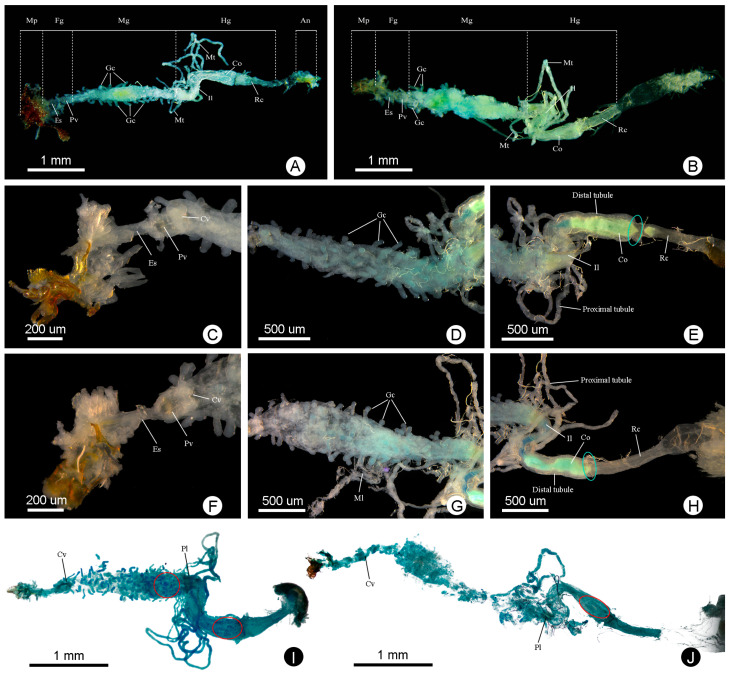
Structural and schematic pictures of the alimentary canal and Malpighian tubules of male and female of *M*. (*O*.) *chinensis*: (**A**) male alimentary canal and Malpighian tubules (LM); (**B**) female alimentary canal and Malpighian tubules (LM); (**C**) male foregut (LM); (**D**) male midgut (LM); (**E**) male hindgut (LM); (**F**) female foregut (LM); (**G**) female midgut (LM); (**H**) female hindgut (LM); (**I**) male alimentary canal and Malpighian tubules (FM); (**J**) female alimentary canal and Malpighian tubules (FM). Green circles in (**E**,**H**) show the short membranous structure. Red circles in (**I**,**J**) show digested pollen grains. LM: light microscopy, FM: fluorescence microscopy, An: anus, Co: colon, Cv: cardiac valve, Es: esophagus, Fg: foregut, Gc: gastric cecum, Hg: hindgut, Il: ileum, Mg: midgut, Ml: muscle layer, Mp: mouthpart, Pl: pylorus region, Pv: proventriculus, Rc: rectum, Mt: Malpighian tubule.

**Figure 4:**
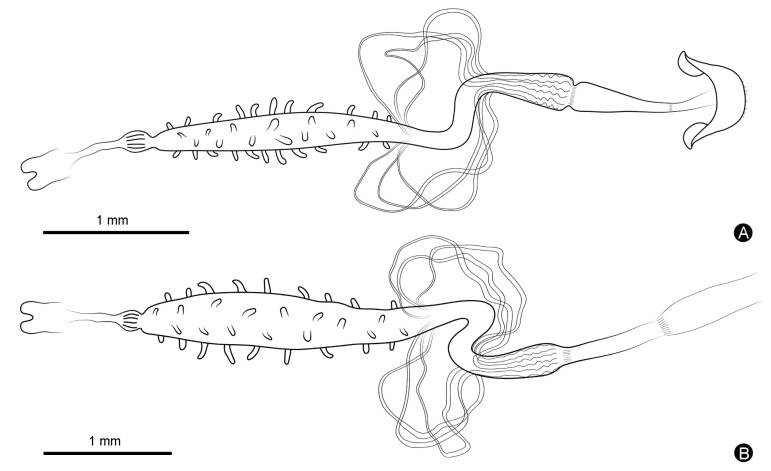
Drawings of the alimentary canal and Malpighian tubules of male and female *M*. (*O*.) *chinensis*: (**A**) male alimentary canal and Malpighian tubules; (**B**) female alimentary canal and Malpighian tubules.

The body length of the randomly selected males of *Meligethes* (*Odonthogethes*) *chinensis* in this study was 3.229 ± 0.033 mm (*n* = 10), and that of females was 3.778 ± 0.094 mm (*n* = 10). Females were, thus, generally larger than males. Correspondingly, females had longer alimentary canals than males of this species. The length of the alimentary canal was 5.264 ± 0.088 mm in males (*n* = 10) and 5.464 ± 0.140 mm in females (*n* = 10). In *M*. (*O*.) *chinensis*, the alimentary canal is tubular, white, and yellow. It consists of three basic parts: the foregut, midgut, and hindgut, which begins with the mouthparts in the head, runs through the thorax, and ends in the anus at the end of the abdomen (Figure 1). The foregut is basically in the head. The midgut extends from the narrow neck to the second abdominal segment. The front of the hindgut folds in a circle and then extends back to the anus. The foregut is the shortest in both males and females. In males, the midgut is the longest. In females, however, the hindgut is the longest (Figure 2). The pyloric region at the junction of the midgut and hindgut, as well as some gut contents (digested pollen grains) in the alimentary canal, can be clearly observed (Figure 3). Six Malpighian tubules are evenly spaced around the alimentary canal, divided into the free tubule region (proximal tubules) and cryptonephridial region (distal tubules). The openings of proximal tubules are inserted into the junction of the midgut and hindgut, and distal tubules form a cryptonephridial system with the colon (Figure 1, Figure 3 and Figure 4).

### 3.2. Foregut

**Figure 5:**
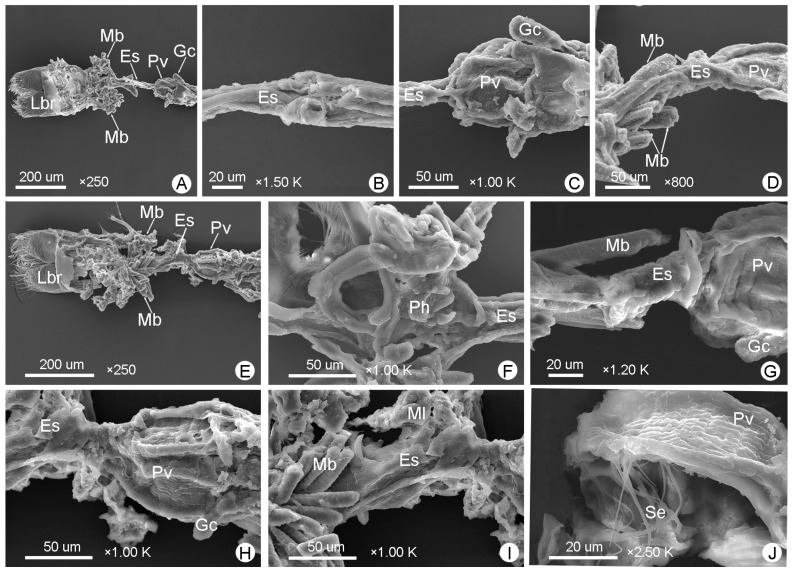
The foreguts of *M*. (*O*.) *chinensis* (males (**A**–**D**), females (**E**–**J**)): (**A**,**E**) general view of the foregut; (**B**,**G**) esophagus (Es); (**C**,**H**) proventriculus (Pv); (**D**,**I**) the muscle bundles (Mb) and muscle layer (Ml) around the foregut; (**F**) pharynx (Ph); (**J**) setae (Se) on the inner surface of the proventriculus. Gc: gastric cecum, Lbr: labrum.

Figure 3C and Figure 5A–D show the foreguts of male *M*. (*O*.) *chinensis*, while Figure 3F and Figure 5E–J show the foreguts of females. The foreguts in males and females are basically the same (Figure 3 and Figure 5). The entire foregut is covered with muscle bundles and muscle layers, almost all located in the head (Figure 5A,D,E,G,I). The foregut is short and consists of the pharynx, esophagus, proventriculus, and cardiac valve (Figure 3 and Figure 5). It connects to the mouthparts and the midgut (Figure 5A,E). The pharynx is a simple channel connecting the mouthparts and the esophagus (Figure 5F). The esophagus is the narrowest part of the whole alimentary canal (Figure 3 and Figure 4; Table 1). It is thin and surrounded by well-defined longitudinal striations and transverse muscles, which can be clearly distinguished from the enlarged proventriculus (Figure 5B,C,G). No crop is found in *M*. (*O*.) *chinensis*. The proventriculus is the widest part of the foregut (Figure 3 and Figure 4; Table 1). It is well developed and is a sclerotized thick-walled, bulbous organ (Figure 5C,G,H,J). On its surface, the longitudinal muscles and transverse muscles, as well as eight proventricular plates, can be clearly observed (Figure 5C,G,H,J). In the longitudinal section, long setae are found on the inner wall of the proventriculus (Figure 5J).

### 3.3. Midgut

**Figure 6:**
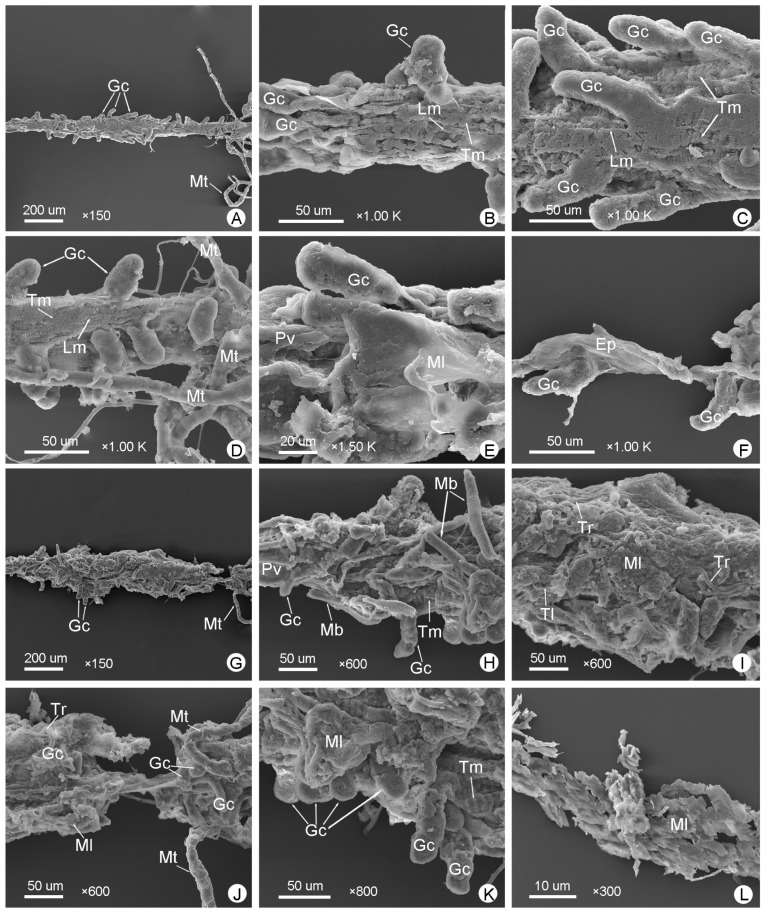
The midguts of *M*. (*O*.) *chinensis* (males (**A**–**F**), females (**G**–**K**)): (**A**,**G**) general view of the midgut; (**B**,**H**) anterior midgut; (**C**,**I**) middle midgut; (**D**,**J**) posterior midgut; (**E**) junction of foregut and midgut; (**F**) epithelium (Ep) of the midgut; (**K**) the muscle layer (Ml) wrapping the midgut; (**L**) part of the muscle layer that wraps the midgut was dissected. Gc: gastric cecum, Lm: longitudinal muscle, Mb: muscle bundle, Mt: Malpighian tubule, Pv: proventriculus, Tl: tracheole, Tm: transverse muscle, Tr: trachea.

Figure 3D and Figure 6A–F show the midguts of male *M*. (*O*.) *chinensis*, while Figure 3G and Figure 6G–K show the midguts of females, and Figure 6L shows part of the muscle layer peeled off around of the female midgut. The midgut is the widest part of the alimentary canal (Figure 3 and Figure 4; Table 1). It is a straight, distended, cylindrical, thin-walled tube that is usually widest in the middle and narrower at the anterior and posterior. Numerous blunt-fingered gastric ceca are distributed irregularly throughout the midgut (Figure 3, Figure 4 and Figure 6A,G). In males, the blunt-fingered gastric ceca can be clearly observed after easily peeling away the muscle layer around of the midgut (Figure 6A–F). The gastric ceca distributed in the middle of the midgut can be longer and larger (Figure 6C), while the gastric ceca on the anterior (Figure 6B,E) and posterior part (Figure 6D) are shorter and smaller. In females, however, it is difficult to observe the gastric ceca directly, because the midgut is tightly wrapped by a thin layer of muscle that is difficult to dissect completely (Figure 3G and Figure 6G–K). It is clearly seen that there are tracheae and tracheoles (Figure 6I,J), as well as even some muscle bundles, covering the anterior part of midgut in females (Figure 6H). Moreover, the transverse muscles and longitudinal muscles, which form a loose network surrounding the midgut, are more clearly observed in males (Figure 6B–D) than in females (Figure 6H,K). The epithelium of the midgut can also be clearly observed after a slight incision on the surface of the midgut (Figure 6F).

### 3.4. Hindgut

**Figure 7:**
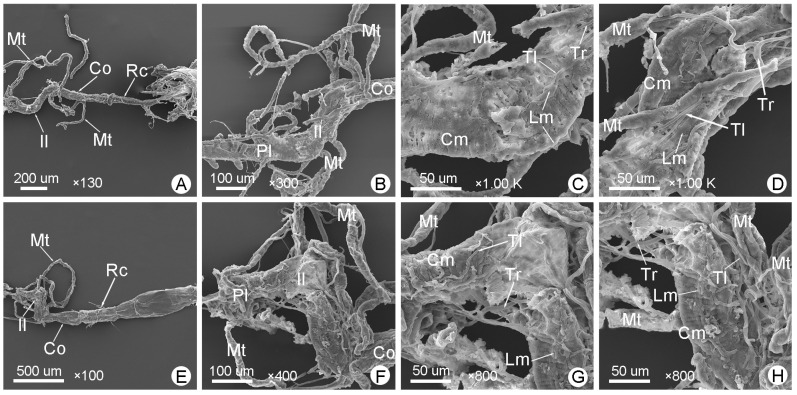
The hindguts of *M*. (*O*.) *chinensis* (males (**A**–**D**), females (**E**–**H**)): (**A**,**E**) general view of the hindgut; (**B**,**F**) pylorus (Pl) and general view of ileum (Il); (**C**,**G**) anterior ileum; (**D**,**H**) posterior ileum. Cm: circular muscle, Co: colon, Lm: longitudinal muscle, Mt: Malpighian tubule, Rc: rectum, Tl: tracheole, Tr: trachea.

**Figure 8:**
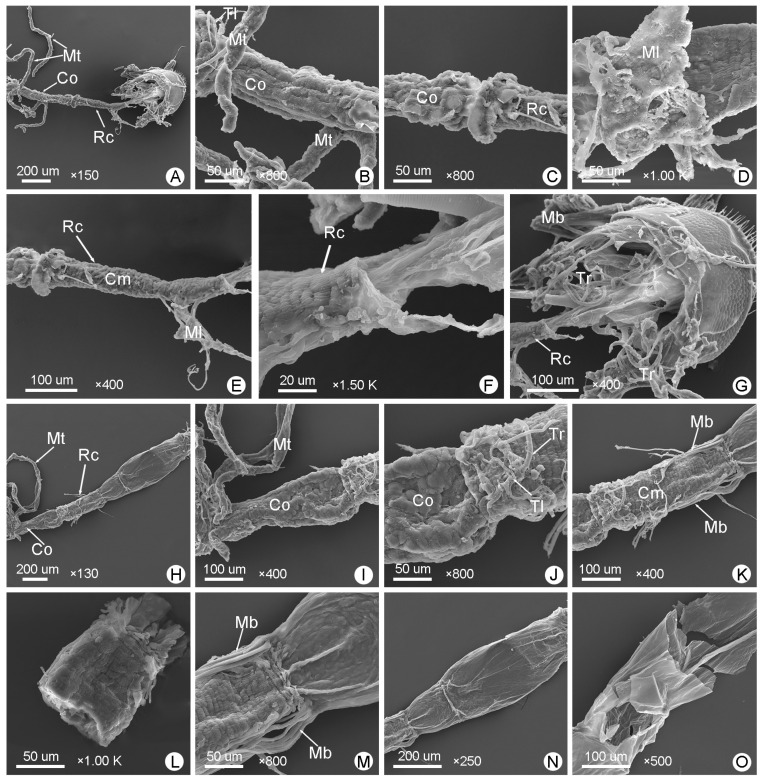
The hindguts and the associated membranous structure of *M*. (*O*.) *chinensis* (males (**A**–**G**), females (**H**–**O**)): (**A**,**H**) general view of colon (Co) and rectum (Rc); (**B**,**I**) general view of colon; (**C**,**J**) junction of colon and rectum; (**D**) the muscle layer (Ml) covering the rectum; (**E**,**K**) circular muscles (Cm) surrounding the rectum; (**F**,**M**) posterior rectum; (**L**) cross-section of rectum; (**G**,**N**,**O**) the membranous structure. Mb: muscle bundle, Mt: Malpighian tubule, Tl: tracheole, Tr: trachea.

The hindgut is coiled. Figure 3E, Figure 7A–D and Figure 8A–F show the hindguts of male *M*. (*O*.) *chinensis*. Figure 3H, Figure 7E–H and Figure 8H–N show the hindguts of females. The hindgut is followed by a membranous structure (Figure 7A,E,G,N,O). The pylorus is at the junction of the midgut and hindgut (Figure 7B,F). The ileum is located between the midgut and colon and is surrounded by free Malpighian tubules. The junction of the midgut and ileum coincides with the opening of the Malpighian tubules, and the junction of the ileum and colon coincides with the end of proximal Malpighian tubules (Figure 7A,B,E,F). The ileum is coiled and densely surrounded by the tracheae and tracheoles. Furthermore, the ileum is surrounded by circular muscles and longitudinal muscles (Figure 3, Figure 4 and Figure 7C,D,G,H). Colon is the widest part of the hindgut (Figure 3 and Figure 4; Table 1). It is straight and progressively enlarged posteriorly, especially in females (Figure 1, Figure 3, Figure 4, Figure 7A,E and Figure 8A,C,H,I). Cryptonephridial region of Malpighian tubules completely attached to the colon, forming irregular deep longitudinal indentations, such that the true colon surface cannot be directly observed (Figure 8B,C,I,J). The rectum is a straight tubular structure, and it is significantly narrower than the colon (Figure 3, Figure 4 and Figure 8A,H; Table 1). It is covered with a layer of muscle that is different from the midgut, surrounded by tracheae and tracheoles (Figure 8D,J). Its surface is surrounded by obvious circular muscles that can be clearly distinguished from the colon (Figure 8B,E,I,K). Between the colon and rectum is a very short membranous structure (Figure 3E,H, Figure 4 and Figure 8C,J). In females, there are muscle bundles on both sides of the distal rectum, elongated and clustered, which differ from the muscle bundles of the foregut (Figure 8K,M). In cross-section, the rectum is quite thick (Figure 8L), followed by a membranous structure (Figure 3, Figure 4 and Figure 8F,M,N,O). In males, this short membranous structure is covered with muscle bundles and connects directly to the anus (Figure 8G). In females, this membranous structure is larger and longer (Figure 8N).

### 3.5. Malpighian Tubules

**Figure 9:**
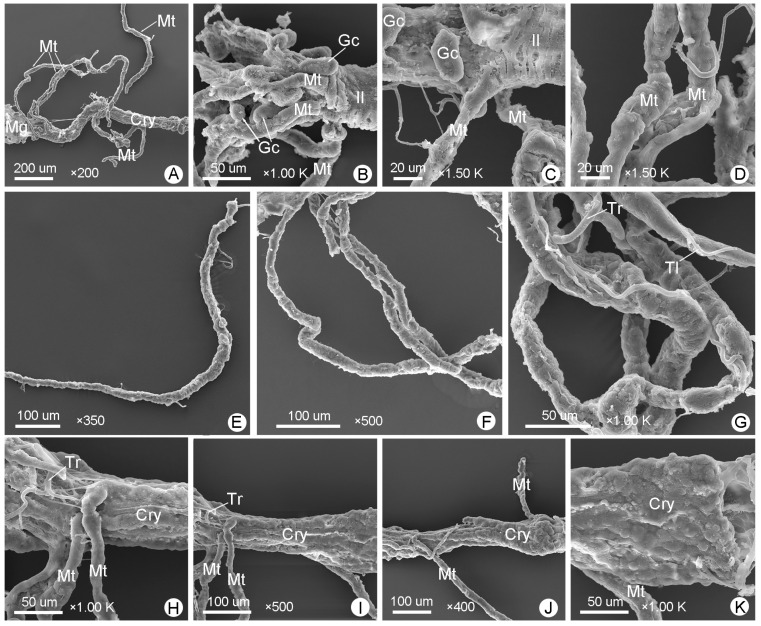
The Malpighian tubules of male *M*. (*O*.) *chinensis*: (**A**) general view of Malpighian tubules (Mt); (**B**,**C**) anterior of free Malpighian tubules; (**D**) posterior of free Malpighian tubules; (**E**) a whole free Malpighian tubule; (**F**) anterior and posterior of free Malpighian tubules present different widths; (**G**) trachea and tracheoles surrounding Malpighian tubules; (**H**–**K**) cryptonephridial system (Cry, distal Malpighian tubules attached to the colon). Gc: gastric cecum, Il: ileum, Mg: midgut, Tl: tracheole, Tr: trachea.

**Figure 10:**
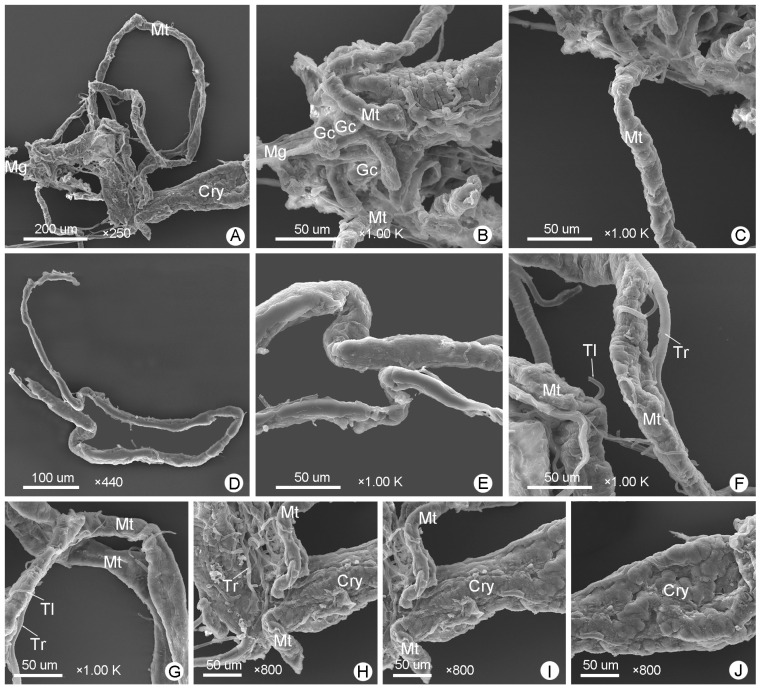
The Malpighian tubules of female *M*. (*O*.) *chinensis*: (**A**) general view of Malpighian tubules (Mt); (**B**,**C**) anterior of free Malpighian tubules; (**D**) a whole free Malpighian tubule; (**E**) anterior and posterior of free Malpighian tubules present different widths; (**F**) trachea and tracheoles surrounding Malpighian tubules; (**G**) posterior of free Malpighian tubules; (**H**–**J**) cryptonephridial system (Cry, distal Malpighian tubules attached to the colon). Gc: gastric cecum, Mg: midgut, Tl: tracheole, Tr: trachea.

*M*. (*O*.) *chinensis* has six Malpighian tubules, each unbranched, nearly equal in length, with an uneven surface, surrounded by tracheae and tracheoles (Figure 9A–K and Figure 10A–J). Figure 9 shows the Malpighian tubules of male *M*. (*O*.) *chinensis*, while Figure 10 shows the Malpighian tubules of female *M*. (*O*.) *chinensis*. Six Malpighian tubules are inserted evenly into the junction of the midgut and hindgut (Figure 9A–C and Figure 10A–C). They can be divided into proximal tubules and distal tubules. Proximal tubules are free in the hemocoelic cavity and surround the posterior of the midgut and the whole ileum, constituting the free tubule region (Figure 9B–H and Figure 10B–H). Each free Malpighian tubule is narrow to wide from the anterior to posterior portion, with the latter being about twice as wide as the former (Figure 9E,F and Figure 10D,E). Distal tubules constituting the cryptonephridial region are completely attached to the colon, forming a cryptonephridial system (Figure 1, Figure 3, Figure 4, Figure 9A,H–K and Figure 10A,H–J). The posterior portion of the distal tubules that attach to the colon is significantly more curved than the anterior (Figure 9I–K and Figure 10H–J). There is no apparent difference in the structure and distribution of Malpighian tubules in male and female *M*. (*O*.) *chinensis* (Figure 9 and Figure 10).

## 4. Discussion

### 4.1. Structural Comparison and Functional Speculation of the Foregut

Beetles are diverse, have complex feeding habits, and have a variety of structures in their alimentary canals [33]. The foregut of *M*. (*O*.) *chinensis* is the shortest part of its alimentary canal, consisting of the pharynx, esophagus, proventriculus, and cardiac valve. Although the foregut is short and narrow, it has a thicker intestinal wall. The pharynx connects the mouth to the esophagus. The esophagus has well-developed striped musculature that may provide energy and impulse for peristalsis [33,67].

#### 4.1.1. The Relationship between Crop Structure and Function and Beetle Feeding Habits

In Coleoptera, the crop is generally absent or only slightly developed in most Polyphaga, while, in Adephaga, the crop is usually present or even well developed [33]. The presence or absence of a crop in the beetle’s foregut is related to its feeding habits and whether there are continuous food sources [33,34,57,68]. The crop is generally considered to have the function of temporarily storing food. Among polyphagous beetles with continuous food sources, Ekis and Gupta (1971) studied 44 species of 22 representative genera in Cleridae and found no crop [50]. This condition was also present in the phytophagous beetles *Aegorhinus supercilious* (Curculionidae) [69], *Eucryptorrhynchus scrobiculatus*, and *E*. *brandti* (Curculionidae) [70]. Concerning the pollen beetle *M*. (*O*.) *chinensis* in this study, no crop was found, probably because *M*. (*O*.) *chinensis* lives in flowers (such as *Rubus idaeus* and other related *Rubus* spp.) with a large amount of pollen as its continuous food source and does not need a crop for temporary food storage.

However, in some phytophagous beetles with continuous food sources, weakly or even well-developed crops were found [32,35,44,58,67,71]. For pollen eaters, well-developed crops filled with pollen grains are present in *Emplesis* and *Misophrice* (Nemonychidae: Erirhininae) (Figure 78 in [42]), as well as *Elleschodes* (Nemonychidae: Tychiinae) (Figure 84 in [42]). Furthermore, two lateral sac-like diverticula with dense pollen grains were described in *Acmaeodera* sp. (Buprestidae) (Figure 79 in [33]), and a lateral sac-like diverticulum is present in *Asclera coerulea* (Oedemeridae) (Figure 149 in [36]). In *A*. *coerulea*, this lateral sac-like diverticulum was observed to have “germinated” pollen grains whose rather tough and impermeable pollen walls were perforated to allow easier access to the pollen grains by digestive enzymes “regurgitated” from the midgut (passed forward from the midgut to the crop) [33,34]. The crop not only serves as a temporary storage site but can also play an important role in the digestion of pollen food [33].

#### 4.1.2. The Relationship between the Structure of the Proventriculus and the Texture of Food

There are obvious differences in the structure and internal organization of the proventriculus among beetles [72]. Nobuchi (1969) studied the structure of the proventriculus of the superfamily Scolytoidea that feed on foods of different textures (phloeophagy, xylomycetophagy, xylophagy, and spermatophagy) and found that proventriculus reduction or anterior plate degeneration occurs in at least three genera (*Gnathotrichus*, *Trypodendron*, and *Xyleborus*) feeding on soft fungi (xylomycetophagy) [37]. Furthermore, Yang et al. (2009) found that *Xylosandrus germanus* (Scolytidae; now Curculionidae Scolitinae), which feeds on fungi (soft texture), has a simple anterior plate [55]. Moreover, the scavenger *Alphitobius diaperinus* (Tenebrionidae) does not have a well-defined proventriculus [73]. The well-developed proventriculus is characteristic of nearly all Adephaga and most Curculionoidea, whereas a weakly developed proventriculus appears in many other Polyphaga [33]. Schedl (1931) emphasized the occurrence of a possible coevolution between feeding habits and the structure of the proventriculus [74].

In *M*. (*O*.) *chinensis* of this study, the proventriculus is the most sclerotized part of the alimentary canal, and the size and development of muscles in this region are increased significantly. The appearance of a well-developed proventriculus seems to be related to the feeding habits of eating hard pollen. Its inner surface is covered with some long setae, similar to plumose setae described in the polyphagous predator *Calosoma sycophanta* (Carabidae) [43], allowing large food particles to be filtered out by the setae.

### 4.2. Structural Comparison, Functional Hypotheses, and Application in the Classification of the Midgut

#### 4.2.1. Study on the Structure and Function of Midgut “Saccate Protrusions”

In morphological studies of the alimentary canal, the terms of Snodgrass (1935) are mainly used. However, as the study of the beetles’ digestive system has become more extensive (increased taxa and advances in techniques), the naming of some structures has become controversial [31]. For example, the “saccate protrusions” on the surface of the midgut of the beetles cannot be clearly distinguished from comparative morphological or functional morphological studies, and the naming is very confusing. In morphological and functional studies of the alimentary canal of different families, such “saccate protrusions” on the midgut have been named papillae, crypts, regenerative crypts, ceca, enteric ceca, gastric ceca, etc.

Gebhardt (1931) claimed that the small, short “papillae” on the surface of the midgut in Buprestidae have a regeneration function [75]. In fact, in many Coleoptera, small papilliform or sometimes elongated diverticula are distributed on the surface of the midgut. Snodgrass (1935) proposed that, in most cases, they were crypts [31]. For example, in *Hydrophilus priceus* (Hydrophilidae), “pouchlike diverticula” formed by evaginations of midgut wall contain epithelial regenerative cells (Figures 202E and 206C,D in [31]), different from true “gastric ceca” in *Dissosteira carolina* (Acrididae) (Figure 195 in [31]); hence, they were named regenerative crypts. In the larvae of lamellicorn beetles *Popillia japonica* (Scarabaeidae), there are three circles of “cecal diverticula” in the midgut, two near the anterior and one near the posterior (Figure 199 in [31]). Bess (1935) claimed that “enteric ceca = crypts” in *Calosoma sycophanta* (Carabidae) [49]. Later, Candan et al. (2020) claimed that “crypts = gastric ceca” in *C*. *sycophanta* [43]. Jaspar-Versali (1987) studied seven carabid species (Carabidae) and proposed that the “gastric ceca” have digestive functions and have regenerative cellular nests [38]. Ameen and Shafiq (1959) proposed that, in Coccinellidae, the columnar cells of the midgut epithelium are replaced by specialized regenerative cells that form deep crypts [76]. In *Adalia bipunctata* (Coccinellidae), there are two circular protrusions at the anterior of midgut, which Borges et al. (2015) called “gastric ceca” [66]. In Curculionidae, Aslam (1961) used “enteric ceca” to describe the finger-like protrusions that are usually located posteriorly [32]. However, Kasap and Crowson (1977) called these similar protrusions “regenerative crypts” in Curculionoidea [77]. Later, Calder (1989) summarized the different situations occurring in Curculionoidea, using “papillae” to describe protrusions that are mostly short or filiform or finger-like with variable distribution [42]. A few and large distinctive pouches distributed on the anterior (or and middle) of the midgut of *Cyrtobagous salviniae* (Figure 79 in [42]), *Notiosomus rugosipennis* (Figure 93 in [42]), and Cossoninae (Figure 94 in [42]) have been called “ceca”. However, it is worth noting that the elongate and narrow protrusions that densely cover the anterior half of the midgut of *Rhabdoscelus* obscurus (Figure 97 in [42]) and the rather short protrusions that densely cover the entire midgut of *Sitophilus linearis* (Figure 98 in [42]) have also been called “ceca”. Thomas (1967) studied 83 species, representing 27 genera of Scolytidae, and he referred to the globular or elongate diverticula as “gastric ceca” [41]. Ekis and Gupta (1971) studied 44 species, representing 22 genera of Cleridae, and they used “papillae” to describe saccate protrusions of different sizes, considering them equivalent to “regenerative crypts” [50]. Crowson (1981) found that small protrusions appear on the surface of the entire midgut of *Acmaeodera* sp. (Buprestidae) (Figure 79 in [33]) and called them “regenerative crypts”. Additionally, in *Dytiscus marginalis* (Dytiscidae) (Figure 78B in [33]), the small pimple-like projections to the long papillae as long as the diameter of the gut itself were all called “regenerative crypts”, bearing small regenerative cells at their apical parts (Figure 3.7b in [78]), called “Nidi”, which were thought to replace the midgut epithelial cells known to have active secretion and absorption functions [33]. In Buprestidae and Elateridae, at the junction of the foregut and midgut, there are appendages or diverticula that are much larger in diameter than the regenerative crypts and have clearly active secretions similar to the general midgut epithelium; these have been called “ceca”, in some cases coexisting with “regenerative crypts” [33]. Crowson (1981) proposed that, even if regenerated crypts are present in adults, they are usually absent at the larval stage [33]. The cecum is usually present in the larvae, tends to be the longest part of the gut of phytophagous larvae, and may have specialized organs, akin to “mycetomes” containing a special symbiote, which may be associated with it, e.g., in *Lixus paraplecticus* (Curculionidae) (Figure 275 in [33]), Silvanidae, Bostrychoidea, and many Cerambycidae. Recently, in Curculionidae, “gastric ceca” of different shapes have been discovered [44,58,71]. For example, in *Epiphaneus malachiticus*, there are short finger-like papillae “gastric ceca” in the middle of the posterior of midgut [71]; in *Tanymecus dilaticollis*, there are eight finger-like “gastric ceca” located distal to the posterior of midgut [58]; *Eusomus ovulum* exhibits numerous short, tubular, cylindrical small pouches referred to as “gastric ceca” on the posterior region of the midgut [44]. Furthermore, numerous small round protrusions called “gastric ceca” on the posterior of midgut were found in *Chrysolina herbacea* (Chrysomelidae) [35]. In *Capnodis tenebrionis* (Buprestidae), Özyurt Koçakoğlu et al. (2020) found a pair of round sacs at the anterior of midgut, called gastric ceca, dorsal-like corn grains and laterally finger-like, coexisting with regularly spaced round regenerative crypts on the surface of the midgut [79]. In *Melanophila picta decastigma* (Buprestidae), Özyurt Koçakoğlu et al. (2021) found a pair of long tubular gastric ceca between the foregut and midgut, coexisting with small round regenerative crypts covering the midgut [45].

Morphologically, there are blunt-fingered saccate protrusions on the surface of the midgut of *Meligethes* (*O*.) *chinensis*, which are similar in shape to the blunt-fingered “gastric ceca” described in the *E*. *malachiticus*, *T*. *dilaticollis*, *E*. *ovulum*, and *C*. *herbacea* [35,44,58,71]. Additionally, the saccate protrusions are irregularly distributed throughout the midgut of *M*. (*O*.) *chinensis*, similar to the distribution of “ceca” in the *R*. *obscurus* and *S*. *linearis* [42]. Therefore, in *M*. (*O*.) *chinensis*, we named these saccate protrusions that spread over the surface of the midgut “gastric ceca”. In the subsequent study of the digestive system of the subfamily Meligethinae, in order to use the terminology more accurately, it is necessary to use “transmission electron microscopy” and “histoenzymology” to further explore the structure and function.

#### 4.2.2. The Application of Midgut “Saccate Protrusions” in Classification

There are usually special saccate protrusions on the midgut of Coleoptera, such as papillae, regenerative crypts, and gastric ceca. These protrusions appear to vary in shape, position, number, and arrangement in different beetles [42,43,71]. Thomas (1967) described and illustrated the midguts of 83 representative species (belonging to 27 genera, 13 tribes, three subfamilies) of Scolytidae, and he found five situations: globular gastric ceca only, elongated gastric ceca only, coexistence of globular and elongated gastric ceca, gastric ceca exhibiting intermediate shape, and absence of gastric ceca. Then, Thomas proposed that the shape of the gastric ceca in different tribes within the same subfamily tends to be consistent; thus, the shape of the gastric ceca can be used as an auxiliary taxonomic characteristic to identify subfamilies. At the genus level, Thomas found that the taxonomically controversial species “*latidens*” had elongated gastric ceca that were more consistent with the genus *Orthotomicus*, thus supporting the assignment of “*latidens*” to the genus *Orthotomicus* rather than to the genus *Ips*. This is good evidence that shows how the shape of the gastric ceca could contribute to the identification of the genus. Even at the species level, Thomas found differences in the number of gastric ceca among these 83 species, but he did not further study whether the number of gastric ceca could be useful for species classification [41]. In the present study, we studied 10 males and 10 females of *M*. (*O*.) *chinensis* and found that the gastric ceca of this species were numerous, blunt-fingered, and distributed irregularly throughout the midgut, similar to *Dendroctonus valens* (Figure 17 in [41]), but quite different from other species of Scolytidae. The shape, position, and arrangement of the gastric ceca are stable in *M*. (*O*.) *chinensis*. However, whether the gastric ceca can be used as a characteristic of the taxonomic identification of Nitidulidae requires further study of more species.

### 4.3. Structural Comparison and Functional Hypotheses of the Hindgut

The structure of the hindgut differs markedly and is segmented and named differently among the different families of Coleoptera. Even within the same family, the structure and segmentation of the hindgut may present differences.

Bess (1935) divided the hindgut of *Calosoma sycophanta* (Carabidae) into three parts: ileum (small intestine), colon, and rectum [49]. Externally, the ileum is indistinguishable from the colon; the rectum is rather large and has six rectal pads. After that, Ali (1964) systematically studied 86 species of 34 representative genera of Carabidae, and then divided the hindgut into two parts, colon and rectum, according to the shape (whether elongated and enlarged); the anterior of rectum was enlarged and presented well-defined rectal glands [80]. Jaspar-Versali et al. (1987) also studied seven carabid species, dividing the hindgut into two parts, ileum and rectum, on the basis of ultrastructural features and the position of rectal pads [38]. Recently, using paraffin sections, as well as hematoxylin and eosin staining, Candan et al. (2020) found that the cross-section of the ileum of *C. sycophanta* is star-shaped, unlike the colon and rectum [43]. Under a high-magnification scanning electron microscope, bacteria and crystals were found in the rectum (Figure 9e,f in [43]). In Chrysomelidae, Davidson (1931) divided the hindgut of *Crioceris asparagi* into three parts: ileum (small intestine), colon, and rectum. Morphologically, the ileum is slender, the colon is gradually enlarged, and the rectum is muscular. Histologically, the intimal folds of the colon are not obvious, the epithelial cells are larger than the ileum, and the rectum has well-defined wavy intimal folds [81]. In Tenebrionidae, Miller (1931) divided the hindgut of *Meracantha contracta* into the ileum (small intestine), colon (large intestine), and rectum according to the thickness and shape of the hindgut. In addition, the distribution of the surface muscle, the folding of the intima, and the shape of the epithelial cells differed among these three sections [82]. Sinha (1958) divided the hindgut of *Tribolium castaneum* into the ileum, colon, and rectum. The cuboid cells of the epithelium in the ileum and rectum are smaller, in contrast to the colon, and the rectum has six rectal pads [83]. Furthermore, Sarwade and Bhawane (2013) used paraffin sections and hematoxylin and eosin staining to compare the muscle development, intimal thickness, and nucleus shape and size in different parts of the hindgut of *Platynotus belli*, thereby dividing the hindgut into the ileum, colon, rectum, and anal canal [84]. In Cerambycidae, Crowson (1981) divided the hindgut of *Cerambyx cerdo* into the small intestine, large intestine, and rectum according to morphology [33]. Yin (1987) dissected 27 species of 21 genera of Cerambycidae, then divided the hindgut into small intestine and rectum according to morphological differences, and further divided the rectum into two distinct parts according to the fitting position of Malpighian tubules [85]. Yin (1996) divided the hindgut of *Philus antennatus* into the small intestine, large intestine, and rectum according to the thickness and shape of the hindgut. The small intestine is slender and curved, and the rectum is straight and thick (Figure 1 in [86]). In Coccinellidae, Potts (1927) divided the hindgut of *Epilachna corrupta* into the ileum, colon, and rectum. The ileum presents a thin intima with only one layer, but the colon and rectum have thick intima with two layers, and the intima of the rectum is jagged-like and more irregular than that of the colon. In addition, the folds of the epithelium, the shape of the nucleus, and the development of circular muscles and longitudinal muscles can be used to distinguish the ileum, colon, and rectum (Figures 16–18 in [87]). Aldigail et al. (2013) divided the hindgut of *Epilachna chrysomelina* into the small intestine (ileum), colon (large intestine), and rectum. The ileum is narrow, the rectum is smooth, transparent, and bladder-like, and the junction of the colon and rectum corresponds to the beginning of distal Malpighian tubules [88]. Borges et al. (2015) divided the hindgut of *Adalia bipunctata* into the ileum, rectum, and rectal canal. The ileum presents six longitudinal folds and a thin circular muscle layer. The rectum and Malpighian tubules form a cryptonephric system, and the epithelium is cubical in the ileum and rectum and squamous in the rectal canal (Figure 5 in [66]). Calder (1989) illustrated 208 species representing 140 genera of Curculionoidea, dividing the hindgut into the ileum, colon, and rectum. Moreover, a sclerotic ring called the rectal valve was found between the colon and rectum [42]. In Curculionoidea and Cleridae, this rectal valve serves as a reference to distinguish the colon from the rectum [42,50]. The rectal valve assists in the excretion of feces (Crowson, 1981) and may play a role in the recovery of water from feces [89]. Candan et al. (2020) divided the hindgut of *Tanymecus dilaticollis* (Curculionidae) into the ileum, colon, and rectum. According to the cross-section, the colon has six large folds that can be distinguished from the ileum and rectum [58]. Özyurt Koçakoğlu et al. (2020) divided the hindgut of *Eusomus ovulum* (Curculionidae) into the ileum, colon, and rectum. From cross-sections of the ileum (Figure 6H in [44]) and colon (Figure 7D in [44]), the lumen size and intimal folding varied, and rectal pads were found in the rectum (Figure 7E in [44]). In Buprestidae, Crowson (1981) divided the hindgut of *Acmaeodera* sp. into the intestine, membranous rectum, and sclerotized rectum according to the morphological characteristics (Figure 79 in [33]). Özyurt Koçakoğlu et al. (2020) divided the hindgut of *Capnodis tenebrionis* into the ileum, colon, and rectum using hematoxylin and eosin staining, light microscopy, and scanning electron microscopy. The surface of the ileum is wavy, and the density of nuclear chromatin in ileal epithelial cells is lower. Malpighian tubules adhere to the surface of the colon, and the nuclei of colonic epithelial cells have dense regions of heterochromatin. Spines were found on the inner surface of the intima of the colon. The rectum has rectal pads, and oval and cubic crystal structures were found in the rectum lumen [79]. Ekis and Gupta (1971) systematically studied 44 species representing 22 genera of Cleridae, and they divided the hindgut into the ileum, colon, and rectum (rectal sac and rectal proper). The intima of the ileum has irregular folds, while the intima of the colon has six larger folds. There is a rectal valve between the colon and rectum. The intima of the rectum differs anteriorly (the rectal sac) and posteriorly (the rectal proper), whereby the rectal sac presents very small folds, while the rectal proper presents long, narrow folds [50].

According to *Crioceris asparagi* (Chrysomelidae) [81], *Epilachna corrupta* (Coccinellidae) (Figures 16–18 in [87]), *Tanymecus dilaticollis* (Curculionidae) [58], and *Eusomus ovulum* (Curculionidae) (Figures 6H and 7D,E in [44]), this study divided the hindgut of pollen beetle *Meligethes* (*O*.) *chinensis* into three parts: ileum, colon, and rectum. The ileum is coiled while the colon gradually enlarges. The ileum and colon can also be distinguished on the basis of surface differences. The circular muscles surrounding the rectum are well developed. Following the rectum of *M*. (*O*.) *chinensis* is a membranous structure connected to the anus, much longer in females than in males, and its unique distensibility appears to be related to excretory function.

### 4.4. Structural Comparison and Functional Hypotheses of the Malpighian Tubules and Cryptonephridial System

The number, distribution, and morphological variation of Malpighian tubules are of great phylogenetic value [50,53]. The original Malpighian tubules of beetles consisted of six tubules that were completely free in the hemocoelic cavity [33,53]. During evolution, Malpighian tubules of some beetles were reduced to four [33,53]. Most Cleridae have six Malpighian tubules, while some species have four [50]. *Holitrica oblita* (Melolonthidae) has four Malpighian tubules [57]. Coleoptera have four or six Malpighian tubules, and they are correspondingly longer [33]. Malpighian tubules are arranged in different ways. The Malpighian tubules are inserted evenly or clustered into two or three groups at the junction of the midgut and hindgut [33,42,52,53]. The posterior proximal tubules are evenly distributed or clustered into two groups or only one group [32,33,52,53]. The distal tubules are mostly evenly attached to the hindgut; however, there are some species, such as in Dermestidae and Bostrychoidea, where all Malpighian tubules are clustered on one side of the hindgut and then form a special protuberance [33,52,53].

During the evolution of beetles, the distal Malpighian tubules (cryptonephridial region) of some species attached to the hindgut, forming a cryptonephridial system [33,53]. The vast majority of these species present each Malpighian tubule attached to the hindgut, whereas, in Apioninae, although six Malpighian tubules occur in some species, only the distal tubules of four are attached to the hindgut [32,52]. Furthermore, in different families of beetles that form a cryptonephridial system, the distal tubules are attached to different parts of the hindgut. In some species, distal tubules are attached to the rectum, forming a cryptonephridial system (Tenebrionidae: Figure 154 in [33]; [54,56]; Figures 1 and 9 in [83]; “P88” in [90]; Scolytidae [55]; Coccinellidae: Figures 1 and 5 in [66]). In some other species, distal tubules are attached to the colon (or large intestine), forming a cryptonephridial system (Chrysomelidae: Figure 9 in [35]; Figure 6 in [81]; Curculionoidea [42]; Carabidae: Figure 8c,e in [43]; Buprestidae: Figure 7 in [45]; Figure 8 in [79]; Cleridae [50]; Curculionidae: Figure 9 in [58]; Figure 13 in [91]; Tenebrionidae: Figure 6 in [73]; Figure 12 in [82]; Cerambycidae: Figure 1 in [86]; Coccinellidae: Figure 17 in [87]; Figure 13 in [88]). In some species, such as *Epiphaneus malachiticus* (Curculionidae: Figure 7 in [71]) and *Eusomus ovulum* (Curculionidae: Figure 7 in [44]), distal tubules are attached to both the colon and the rectum, forming a cryptonephridial system.

In *M*. (*O*.) *chinensis*, according to LM, FM, and SEM studies, proximal tubules are free and evenly spaced in the hemocoelic cavity and surround the posterior of the midgut and the whole ileum, while distal tubules are evenly attached to the colon, forming a cryptonephridial system, consistent with the Malpighian tubules of *Meligethes* drawn by Stammer (1934) in Figure 12 based on LM [52].

## 5. Conclusions

In this paper, we studied the morphology of the alimentary canal and Malpighian tubules of the pollen beetle *M*. (*O*.) *chinensis*, whose main host plant is *Rubus idaeus* L. (Rosaceae). For the first time, the fine morphological structure of the alimentary canal and Malpighian tubules of pollen beetle Meligethinae was comprehensively revealed. Special structural features were identified such as the absence of crop, setae on the inner surface of the sclerotized proventriculus, numerous blunt-fingered gastric ceca throughout the midgut, and the openings of six Malpighian tubules inserted evenly into the junction of the midgut and hindgut and distal tubules attached to the colon. We need to further study the comparative morphology of the alimentary canal and Malpighian tubules of Meligethinae using TEM, micro-CT, etc. to reveal the pollen digestion and absorption function of pollen beetles, as well as to provide a basis for the coevolution of pollen beetles and host plants, in addition to taxonomic, evolutionary, and phylogenetic studies of Coleoptera.

## Figures and Tables

**Table 1:** The width (mm) (mean ± SE) of different organs in the alimentary canal of male and female *M*. (*O*.) *chinensis*.

Organ	Organ Width (Mean ± SE) (mm)		Student’s *t*-Test (*t*, *p*) or Mann–Whitney Test (Z, *p*)
	Male	Female	
Esophagus	0.074 ± 0.005 a	0.075 ± 0.006 a	*t* = −0.132, *p* = 0.897
Proventriculus	0.152 ± 0.013 a	0.174 ± 0.009 a	*Z =* −1.440, *p =* 0.150
Midgut	0.372 ± 0.029 a	0.422 ± 0.028 a	*Z* = −1.437, *p* = 0.151
Ileum	0.164 ± 0.012 a	0.139 ± 0.009 a	*Z* = −1.551, *p* = 0.121
Colon	0.231 ± 0.015 a	0.227 ± 0.019 a	*t* = 0.179, *p* = 0.860
Rectum	0.170 ± 0.010 a	0.151 ± 0.008 a	*t* = 1.449, *p* = 0.165

Note: In the same row, the same letter (a, a) indicates no significant difference (*n* = 10).

## Data Availability

The data presented in this study are available on request from the corresponding author.
